# Unveiling the hidden burden: the impact of undiagnosed comorbidities on health-related quality of life in ICU survivors

**DOI:** 10.1186/s13054-024-05025-8

**Published:** 2024-07-10

**Authors:** Jill Moser, Roos Mensink, Marisa Onrust, Fredrike Blokzijl, Jacqueline Koeze

**Affiliations:** Department of Critical Care, University of Groningen, University Medical Center Groningen, Groningen, The Netherlands

## To the Editor,

With great interest we read the recent article by Orwelius et al. [1] on the impact of comorbidities on health-related quality of life (HRQoL) in ICU survivors. We commend the authors for their work on addressing this challenging topic and for providing an insightful review. While we agree with the key issues highlighted by the authors, we believe that several points warrant further discussion.

One of the major challenges in assessing the impact of comorbidities on HRQoL among ICU survivors is obtaining accurate comorbidity prevalence data, both at ICU admission and during post-ICU follow-up. ICU physicians rely on medical records to identify patient comorbidities; however, conditions such as hypertension, diabetes, and chronic kidney disease (CKD) frequently remain undiagnosed until they reach advanced stages. For instance, elevated glucose levels are commonly found in patients admitted to the ICU, despite having no medical history of diabetes. While this is typically attributed to critical illness, it may also be partly due to underlying diabetes or prediabetes. Similarly, baseline serum creatinine levels are often unknown upon ICU admission. As a result, elevated creatinine levels may be misinterpreted as acute kidney injury when it could partly stem from pre-existing CKD. Our recent findings underscore this issue, revealing that while 17% of hospitalized COVID-19 patients had a medical history of CKD, 93% exhibited renal histopathological features consistent with the disease [2]. Hence, the underdiagnosis of chronic conditions can lead to an underestimation of the true burden and severity of comorbidities in patients admitted to the ICU. Consequently, the impact of these conditions on HRQoL might be inaccurately attributed solely to ICU stay when, in reality, pre-existing but undiagnosed conditions play a substantial role.

In addition, there are notable sex differences in the reporting and management of chronic health issues. Men and women may experience and report comorbidities differently and may also have different health-seeking behaviors [3]. Women, for instance, are more likely to seek medical care and thus may have their chronic conditions diagnosed earlier and managed more effectively than men. This disparity can lead to biased data regarding the prevalence and impact of comorbidities on HRQoL.

Similar to the observations of Orwelius et al., we emphasize that using age adjustment in HRQoL studies to account for comorbidities is insufficient. In recent years, there has been a noticeable shift in the age at which comorbidities are being diagnosed, with younger populations increasingly exhibiting chronic diseases traditionally associated with older adults. This trend is largely due to unhealthy lifestyle choices prevalent in modern society, such as poor dietary habits, physical inactivity, and elevated stress levels. Consequently, conditions such as hypertension, type 2 diabetes, and liver disease are now being diagnosed in younger individuals, highlighting the need for more nuanced approaches to HRQoL research.

It is well documented that critical illness can often lead to chronic conditions post-ICU, such as progression from acute kidney injury to chronic kidney disease [4, 5]. Increasing evidence suggests that other chronic diseases, like diabetes, may also develop following an ICU stay [6, 7]; however, this phenomenon remains understudied. This research gap is partly due to the lack of structured and accurate diagnosis of chronic disease and their severity at ICU admission and during post-ICU follow-up. New-onset chronic disease or exacerbation of pre-existing conditions will undoubtedly have a significant impact on HRQoL. Therefore, early identification of chronic disease will lead to more effective management strategies, improve patient recovery, and ultimately HRQoL, as well as reduce the burden on healthcare systems.

The review by Orwelius et al., importantly underscores the necessity for standardized, comprehensive approaches to comorbidity assessment in future research. The heterogeneity in HRQoL measurement tools and follow-up durations across the studies reviewed makes it challenging to draw definitive conclusions about the true impact of comorbidities versus the ICU stay itself. For instance, the use of different tools, such as SF-36, EQ-5D, and others, each with their own strengths and limitations, leads to variability in the reported outcomes. Furthermore, the absence of control groups adequately matched for undiagnosed and likely uncontrolled or early stage comorbidities limits the ability to isolate the specific effects of ICU stay on HRQoL. Moreover, the impact of non-ICU-related factors on HRQoL is usually overlooked, with potential unmeasured confounders existing, regardless of ICU admission. A standardized approach to measuring HRQoL, coupled with thorough and consistent comorbidity assessments that may require laboratory tests, would enhance the comparability and reliability of future studies.

In conclusion, addressing inaccuracies in comorbidity prevalence data, considering sex differences in health reporting and management, and adopting standardized HRQoL assessment methods are crucial. Moreover, implementing laboratory assessments of blood and/or urine samples to accurately identify comorbidities at ICU admission and during post-ICU follow-up is essential. Future research must incorporate these measures to provide a more accurate and comprehensive understanding of comorbidities and other factors influencing HRQoL in ICU survivors.

## Data Availability

Not applicable.

## Abbreviations

ICU: Intensive care unit
HRQoL: Health related quality of life
CKD: Chronic kidney disease
SF-36: Short form 36 questions
EQ-5D: EuroQol 5 dimensions

## References

[1] Orwelius L, Wilhelms S, Sjöberg F (2024). Is comorbidity alone responsible for changes in health-related quality of life among critical care survivors? A purpose-specific review. Crit Care.

[2] Volbeda M, Jou-Valencia D, van den Heuvel MC, Moser J, van Meurs M, Franssen CFM (2023). Letter regarding “association between CKD, obesity, cardiometabolic risk factors, and severe COVID-19 outcomes”. Kidney Int Rep.

[3] Thompson AE, Anisimowicz Y, Miedema B, Hogg W, Wodchis WP, Aubrey-Bassler K (2016). The influence of gender and other patient characteristics on health care-seeking behaviour: a QUALICOPC study. BMC Fam Pract.

[4] See EJ, Jayasinghe K, Glassford N, Bailey M, Johnson DW, Polkinghorne KR (2019). Long-term risk of adverse outcomes after acute kidney injury: a systematic review and meta-analysis of cohort studies using consensus definitions of exposure. Kidney Int.

[5] Fiorentino M, Grandaliano G, Gesualdo L, Castellano G (2018). Acute kidney injury to chronic kidney disease transition. Acute Kidney Injury Basic Res Clin Pract.

[6] Ali Abdelhamid Y, Kar P, Finnis ME, Phillips LK, Plummer MP, Shaw JE (2016). Stress hyperglycaemia in critically ill patients and the subsequent risk of diabetes: a systematic review and meta-analysis. Crit Care.

[7] Jivanji CJ, Asrani VM, Windsor JA, Petrov MS (2017). New-onset diabetes after acute and critical illness: a systematic review. Mayo Clin Proc.

